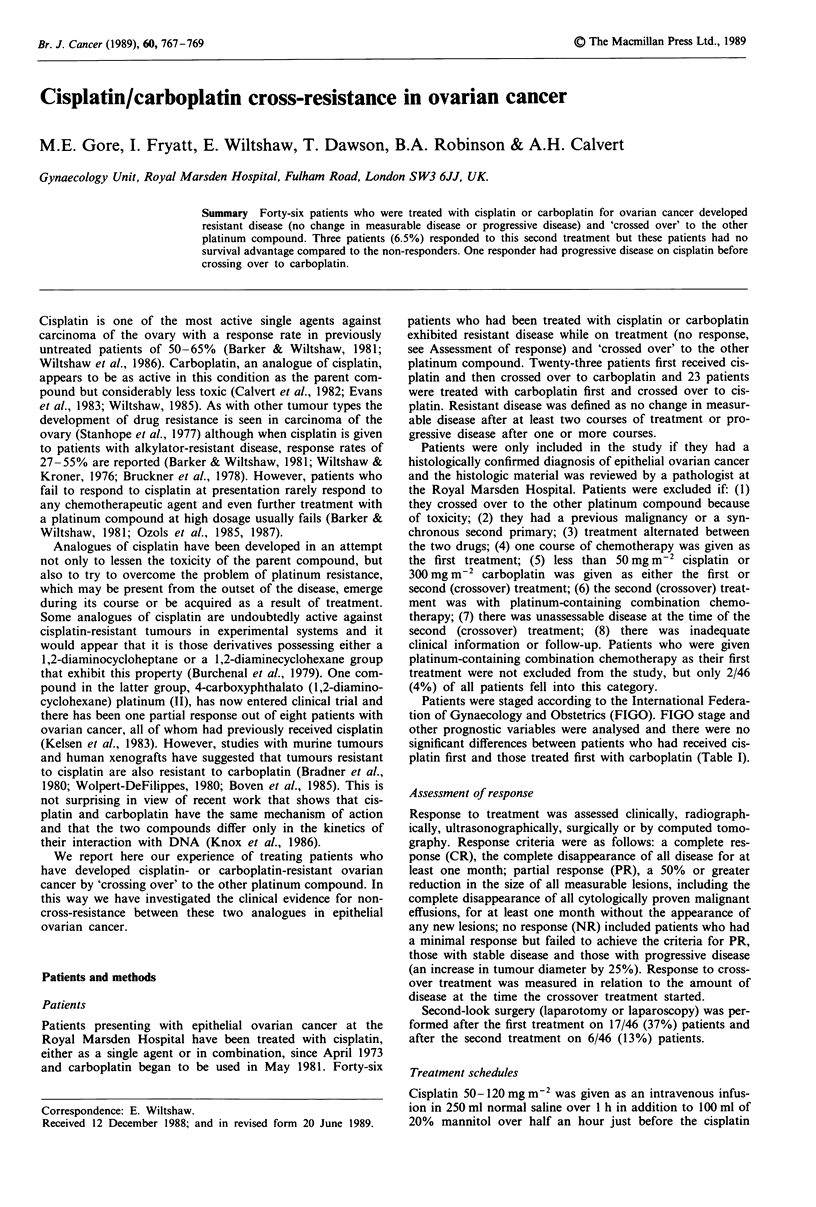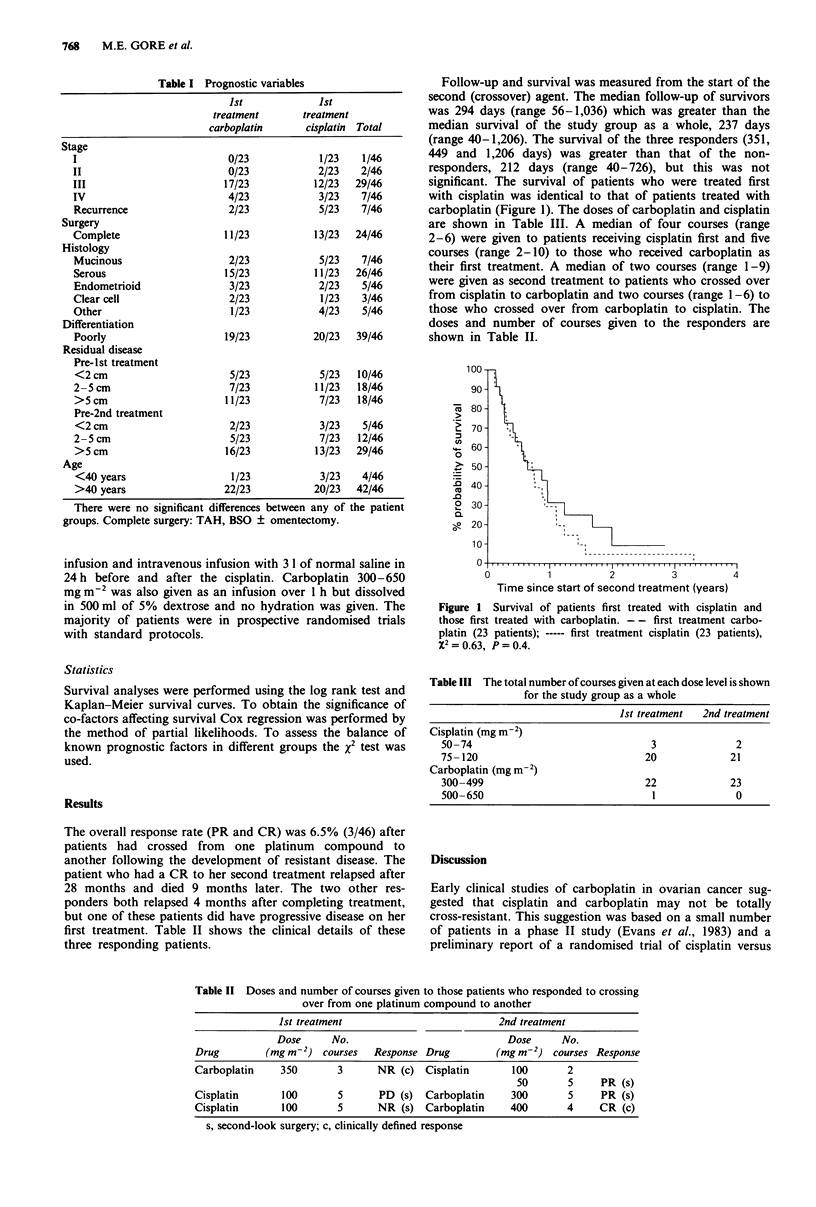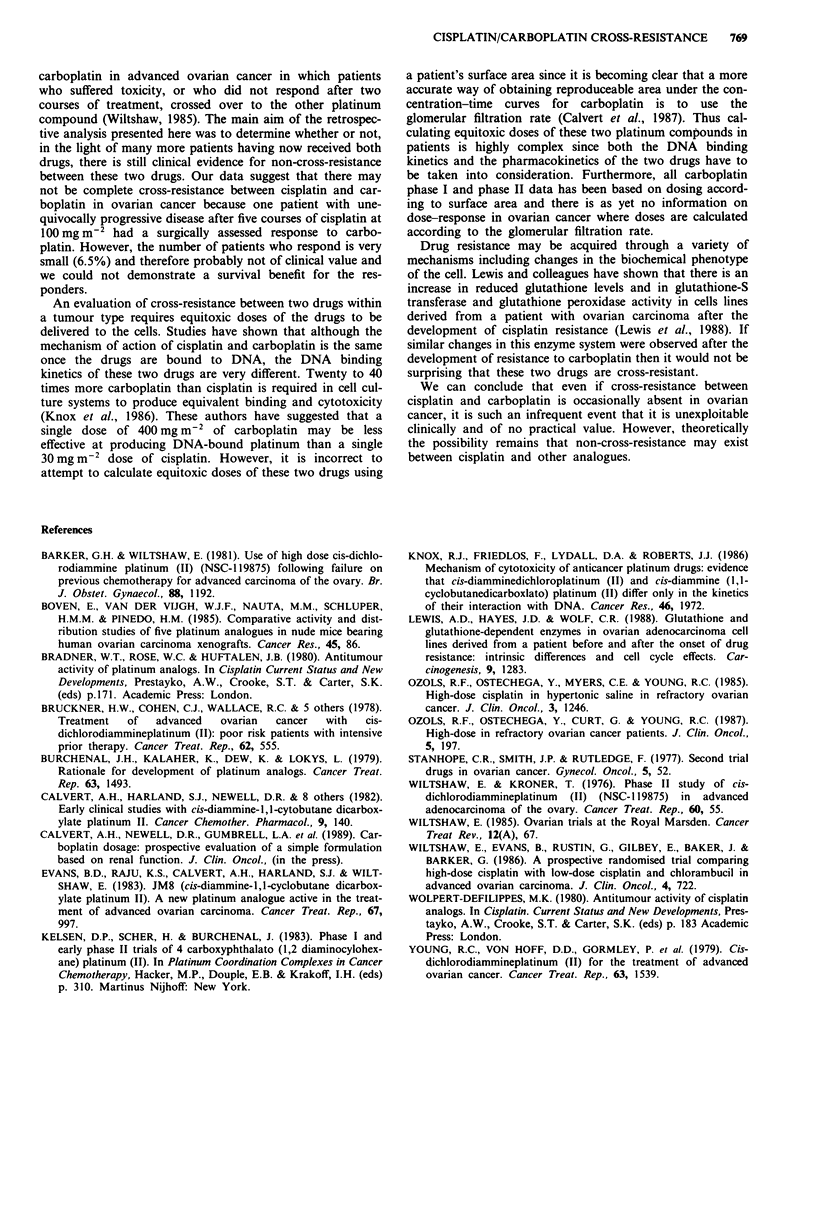# Cisplatin/carboplatin cross-resistance in ovarian cancer.

**DOI:** 10.1038/bjc.1989.356

**Published:** 1989-11

**Authors:** M. E. Gore, I. Fryatt, E. Wiltshaw, T. Dawson, B. A. Robinson, A. H. Calvert

**Affiliations:** Gynaecology Unit, Royal Marsden Hospital, London, UK.

## Abstract

Forty-six patients who were treated with cisplatin or carboplatin for ovarian cancer developed resistant disease (no change in measurable disease or progressive disease) and 'crossed over' to the other platinum compound. Three patients (6.5%) responded to this second treatment but these patients had no survival advantage compared to the non-responders. One responder had progressive disease on cisplatin before crossing over to carboplatin.


Br. J. Cancer (1989), 60, 767–769 © The Macmillan Press Ltd., 1989

### Cisplatin/carboplatin cross-resistance in ovarian cancer

M.E. Gore, I. Fryatt, E. Wiltshaw, T. Dawson, B.A. Robinson & A.H. Calvert

*Gynaecology Unit, Royal Marsden Hospital, Fulham Road, London SW3 6JJ, UK.*

**Summary** Forty-six patients who were treated with cisplatin or carboplatin for ovarian cancer developed resistant disease (no change in measurable disease or progressive disease) and 'crossed over' to the other platinum compound. Three patients (6.5%) responded to this second treatment but these patients had no survival advantage compared to the non-responders. One responder had progressive disease on cisplatin before crossing over to carboplatin.

Cisplatin is one of the most active single agents against carcinoma of the ovary with a response rate in previously untreated patients of 50–65% (Barker & Wiltshaw, 1981; Wiltshaw *et al.*, 1986). Carboplatin, an analogue of cisplatin, appears to be as active in this condition as the parent compound but considerably less toxic (Calvert *et al.*, 1982; Evans *et al.*, 1983; Wiltshaw, 1985). As with other tumour types the development of drug resistance is seen in carcinoma of the ovary (Stanhope *et al.*, 1977) although when cisplatin is given to patients with alkylator-resistant disease, response rates of 27–55% are reported (Barker & Wiltshaw, 1981; Wiltshaw & Kroner, 1976; Bruckner *et al.*, 1978). However, patients who fail to respond to cisplatin at presentation rarely respond to any chemotherapeutic agent and even further treatment with a platinum compound at high dosage usually fails (Barker & Wiltshaw, 1981; Ozols *et al.*, 1985, 1987).

Analogues of cisplatin have been developed in an attempt not only to lessen the toxicity of the parent compound, but also to try to overcome the problem of platinum resistance, which may be present from the outset of the disease, emerge during its course or be acquired as a result of treatment. Some analogues of cisplatin are undoubtedly active against cisplatin-resistant tumours in experimental systems and it would appear that it is those derivatives possessing either a 1,2-diaminocycloheptane or a 1,2-diaminecyclohexane group that exhibit this property (Burchenal *et al.*, 1979). One compound in the latter group, 4-carboxyphthalato (1,2-diaminocyclohexane) platinum (II), has now entered clinical trial and there has been one partial response out of eight patients with ovarian cancer, all of whom had previously received cisplatin (Kelsen *et al.*, 1983). However, studies with murine tumours and human xenografts have suggested that tumours resistant to cisplatin are also resistant to carboplatin (Bradner *et al.*, 1980; Wolpert-DeFilippes, 1980; Boven *et al.*, 1985). This is not surprising in view of recent work that shows that cisplatin and carboplatin have the same mechanism of action and that the two compounds differ only in the kinetics of their interaction with DNA (Knox *et al.*, 1986).

We report here our experience of treating patients who have developed cisplatin- or carboplatin-resistant ovarian cancer by 'crossing over' to the other platinum compound. In this way we have investigated the clinical evidence for non-cross-resistance between these two analogues in epithelial ovarian cancer.

### Patients and methods

### *Patients*

Patients presenting with epithelial ovarian cancer at the Royal Marsden Hospital have been treated with cisplatin, either as a single agent or in combination, since April 1973 and carboplatin began to be used in May 1981. Forty-six patients who had been treated with cisplatin or carboplatin exhibited resistant disease while on treatment (no response, see Assessment of response) and 'crossed over' to the other platinum compound. Twenty-three patients first received cisplatin and then crossed over to carboplatin and 23 patients were treated with carboplatin first and crossed over to cisplatin. Resistant disease was defined as no change in measurable disease after at least two courses of treatment or progressive disease after one or more courses.

Patients were only included in the study if they had a histologically confirmed diagnosis of epithelial ovarian cancer and the histologic material was reviewed by a pathologist at the Royal Marsden Hospital. Patients were excluded if: (1) they crossed over to the other platinum compound because of toxicity; (2) they had a previous malignancy or a synchronous second primary; (3) treatment alternated between the two drugs; (4) one course of chemotherapy was given as the first treatment; (5) less than 50 mg m$^{-2}$ cisplatin or 300 mg m$^{-2}$ carboplatin was given as either the first or second (crossover) treatment; (6) the second (crossover) treatment was with platinum-containing combination chemotherapy; (7) there was unassessable disease at the time of the second (crossover) treatment; (8) there was inadequate clinical information or follow-up. Patients who were given platinum-containing combination chemotherapy as their first treatment were not excluded from the study, but only 2/46 (4%) of all patients fell into this category.

Patients were staged according to the International Federation of Gynaecology and Obstetrics (FIGO). FIGO stage and other prognostic variables were analysed and there were no significant differences between patients who had received cisplatin first and those treated first with carboplatin (Table I).

### *Assessment of response*

Response to treatment was assessed clinically, radiographically, ultrasonographically, surgically or by computed tomography. Response criteria were as follows: a complete response (CR), the complete disappearance of all disease for at least one month; partial response (PR), a 50% or greater reduction in the size of all measurable lesions, including the complete disappearance of all cytologically proven malignant effusions, for at least one month without the appearance of any new lesions; no response (NR) included patients who had a minimal response but failed to achieve the criteria for PR, those with stable disease and those with progressive disease (an increase in tumour diameter by 25%). Response to crossover treatment was measured in relation to the amount of disease at the time the crossover treatment started.

Second-look surgery (laparotomy or laparoscopy) was performed after the first treatment on 17/46 (37%) patients and after the second treatment on 6/46 (13%) patients.

### *Treatment schedules*

Cisplatin 50–120 mg m$^{-2}$ was given as an intravenous infusion in 250 ml normal saline over 1 h in addition to 100 ml of 20% mannitol over half an hour just before the cisplatin

Correspondence: E. Wiltshaw.
Received 12 December 1988; and in revised form 20 June 1989.

**Table I** Prognostic variables

| | 1st treatment carboplatin | 1st treatment cisplatin | Total |
|---|---|---|---|
| Stage | | | |
| I | 0/23 | 1/23 | 1/46 |
| II | 0/23 | 2/23 | 2/46 |
| III | 17/23 | 12/23 | 29/46 |
| IV | 4/23 | 3/23 | 7/46 |
| Recurrence | 2/23 | 5/23 | 7/46 |
| Surgery | | | |
| Complete | 11/23 | 13/23 | 24/46 |
| Histology | | | |
| Mucinous | 2/23 | 5/23 | 7/46 |
| Serous | 15/23 | 11/23 | 26/46 |
| Endometrioid | 3/23 | 2/23 | 5/46 |
| Clear cell | 2/23 | 1/23 | 3/46 |
| Other | 1/23 | 4/23 | 5/46 |
| Differentiation | | | |
| Poorly | 19/23 | 20/23 | 39/46 |
| Residual disease | | | |
| Pre-1st treatment | | | |
| <2 cm | 5/23 | 5/23 | 10/46 |
| 2-5 cm | 7/23 | 11/23 | 18/46 |
| >5 cm | 11/23 | 7/23 | 18/46 |
| Pre-2nd treatment | | | |
| <2 cm | 2/23 | 3/23 | 5/46 |
| 2-5 cm | 5/23 | 7/23 | 12/46 |
| >5 cm | 16/23 | 13/23 | 29/46 |
| Age | | | |
| <40 years | 1/23 | 3/23 | 4/46 |
| >40 years | 22/23 | 20/23 | 42/46 |

There were no significant differences between any of the patient groups. Complete surgery: TAH, BSO ± omentectomy.

infusion and intravenous infusion with 3 l of normal saline in 24 h before and after the cisplatin. Carboplatin 300–650 mg m$^{-2}$ was also given as an infusion over 1 h but dissolved in 500 ml of 5% dextrose and no hydration was given. The majority of patients were in prospective randomised trials with standard protocols.

### *Statistics*

Survival analyses were performed using the log rank test and Kaplan–Meier survival curves. To obtain the significance of co-factors affecting survival Cox regression was performed by the method of partial likelihoods. To assess the balance of known prognostic factors in different groups the $\chi^2$ test was used.

### Results

The overall response rate (PR and CR) was 6.5% (3/46) after patients had crossed from one platinum compound to another following the development of resistant disease. The patient who had a CR to her second treatment relapsed after 28 months and died 9 months later. The two other responders both relapsed 4 months after completing treatment, but one of these patients did have progressive disease on her first treatment. Table II shows the clinical details of these three responding patients.

Follow-up and survival was measured from the start of the second (crossover) agent. The median follow-up of survivors was 294 days (range 56–1,036) which was greater than the median survival of the study group as a whole, 237 days (range 40–1,206). The survival of the three responders (351, 449 and 1,206 days) was greater than that of the non-responders, 212 days (range 40–726), but this was not significant. The survival of patients who were treated first with cisplatin was identical to that of patients treated with carboplatin (Figure 1). The doses of carboplatin and cisplatin are shown in Table III. A median of four courses (range 2–6) were given to patients receiving cisplatin first and five courses (range 2–10) to those who received carboplatin as their first treatment. A median of two courses (range 1–9) were given as second treatment to patients who crossed over from cisplatin to carboplatin and two courses (range 1–6) to those who crossed over from carboplatin to cisplatin. The doses and number of courses given to the responders are shown in Table II.

**Figure 1** Survival of patients first treated with cisplatin and those first treated with carboplatin. — — first treatment carboplatin (23 patients); ----- first treatment cisplatin (23 patients), $\chi^2 = 0.63$, $P = 0.4$.

**Table III** The total number of courses given at each dose level is shown for the study group as a whole

| | 1st treatment | 2nd treatment |
|---|---|---|
| Cisplatin (mg m$^{-2}$) | | |
| 50–74 | 3 | 2 |
| 75–120 | 20 | 21 |
| Carboplatin (mg m$^{-2}$) | | |
| 300–499 | 22 | 23 |
| 500–650 | 1 | 0 |

### Discussion

Early clinical studies of carboplatin in ovarian cancer suggested that cisplatin and carboplatin may not be totally cross-resistant. This suggestion was based on a small number of patients in a phase II study (Evans *et al.*, 1983) and a preliminary report of a randomised trial of cisplatin versus

**Table II** Doses and number of courses given to those patients who responded to crossing over from one platinum compound to another

| 1st treatment | | | | 2nd treatment | | | |
|---|---|---|---|---|---|---|---|
| Drug | Dose (mg m$^{-2}$) | No. courses | Response | Drug | Dose (mg m$^{-2}$) | No. courses | Response |
| Carboplatin | 350 | 3 | NR (c) | Cisplatin | 100 | 2 | |
| | | | | | 50 | 5 | PR (s) |
| Cisplatin | 100 | 5 | PD (s) | Carboplatin | 300 | 5 | PR (s) |
| Cisplatin | 100 | 5 | NR (s) | Carboplatin | 400 | 4 | CR (c) |

s, second-look surgery; c, clinically defined response carboplatin in advanced ovarian cancer in which patients who suffered toxicity, or who did not respond after two courses of treatment, crossed over to the other platinum compound (Wiltshaw, 1985). The main aim of the retrospective analysis presented here was to determine whether or not, in the light of many more patients having now received both drugs, there is still clinical evidence for non-cross-resistance between these two drugs. Our data suggest that there may not be complete cross-resistance between cisplatin and carboplatin in ovarian cancer because one patient with unequivocally progressive disease after five courses of cisplatin at $100\,mg\,m^{-2}$ had a surgically assessed response to carboplatin. However, the number of patients who respond is very small (6.5%) and therefore probably not of clinical value and we could not demonstrate a survival benefit for the responders.

An evaluation of cross-resistance between two drugs within a tumour type requires equitoxic doses of the drugs to be delivered to the cells. Studies have shown that although the mechanism of action of cisplatin and carboplatin is the same once the drugs are bound to DNA, the DNA binding kinetics of these two drugs are very different. Twenty to 40 times more carboplatin than cisplatin is required in cell culture systems to produce equivalent binding and cytotoxicity (Knox *et al.*, 1986). These authors have suggested that a single dose of $400\,mg\,m^{-2}$ of carboplatin may be less effective at producing DNA-bound platinum than a single $30\,mg\,m^{-2}$ dose of cisplatin. However, it is incorrect to attempt to calculate equitoxic doses of these two drugs using a patient's surface area since it is becoming clear that a more accurate way of obtaining reproduceable area under the concentration–time curves for carboplatin is to use the glomerular filtration rate (Calvert *et al.*, 1987). Thus calculating equitoxic doses of these two platinum compounds in patients is highly complex since both the DNA binding kinetics and the pharmacokinetics of the two drugs have to be taken into consideration. Furthermore, all carboplatin phase I and phase II data has been based on dosing according to surface area and there is as yet no information on dose–response in ovarian cancer where doses are calculated according to the glomerular filtration rate.

Drug resistance may be acquired through a variety of mechanisms including changes in the biochemical phenotype of the cell. Lewis and colleagues have shown that there is an increase in reduced glutathione levels and in glutathione-S transferase and glutathione peroxidase activity in cells lines derived from a patient with ovarian carcinoma after the development of cisplatin resistance (Lewis *et al.*, 1988). If similar changes in this enzyme system were observed after the development of resistance to carboplatin then it would not be surprising that these two drugs are cross-resistant.

We can conclude that even if cross-resistance between cisplatin and carboplatin is occasionally absent in ovarian cancer, it is such an infrequent event that it is unexploitable clinically and of no practical value. However, theoretically the possibility remains that non-cross-resistance may exist between cisplatin and other analogues.

### References

BARKER, G.H. & WILTSHAW, E. (1981). Use of high dose cis-dichlorodiammine platinum (II) (NSC-119875) following failure on previous chemotherapy for advanced carcinoma of the ovary. *Br. J. Obstet. Gynaecol.*, **88**, 1192.

BOVEN, E., VAN DER VIJGH, W.J.F., NAUTA, M.M., SCHLUPER, H.M.M. & PINEDO, H.M. (1985). Comparative activity and distribution studies of five platinum analogues in nude mice bearing human ovarian carcinoma xenografts. *Cancer Res.*, **45**, 86.

BRADNER, W.T., ROSE, W.C. & HUFTALEN, J.B. (1980). Antitumour activity of platinum analogs. In *Cisplatin Current Status and New Developments*, Prestayko, A.W., Crooke, S.T. & Carter, S.K. (eds) p.171. Academic Press: London.

BRUCKNER, H.W., COHEN, C.J., WALLACE, R.C. & 5 others (1978). Treatment of advanced ovarian cancer with cis-dichlorodiammineplatinum (II): poor risk patients with intensive prior therapy. *Cancer Treat. Rep.*, **62**, 555.

BURCHENAL, J.H., KALAHER, K., DEW, K. & LOKYS, L. (1979). Rationale for development of platinum analogs. *Cancer Treat. Rep.* **63**, 1493.

CALVERT, A.H., HARLAND, S.J., NEWELL, D.R. & 8 others (1982). Early clinical studies with *cis*-diammine-1,1-cytobutane dicarboxylate platinum II. *Cancer Chemother. Pharmacol.*, **9**, 140.

CALVERT, A.H., NEWELL, D.R., GUMBRELL, L.A. *et al.* (1989). Carboplatin dosage: prospective evaluation of a simple formulation based on renal function. *J. Clin. Oncol.*, (in the press).

EVANS, B.D., RAJU, K.S., CALVERT, A.H., HARLAND, S.J. & WILTSHAW, E. (1983). JM8 (*cis*-diammine-1,1-cyclobutane dicarboxylate platinum II). A new platinum analogue active in the treatment of advanced ovarian carcinoma. *Cancer Treat. Rep.*, **67**, 997.

KELSEN, D.P., SCHER, H. & BURCHENAL, J. (1983). Phase I and early phase II trials of 4 carboxyphthalato (1,2 diaminocylohexane) platinum (II). In *Platinum Coordination Complexes in Cancer Chemotherapy*, Hacker, M.P., Douple, E.B. & Krakoff, I.H. (eds) p. 310. Martinus Nijhoff: New York.

KNOX, R.J., FRIEDLOS, F., LYDALL, D.A. & ROBERTS, J.J. (1986) Mechanism of cytotoxicity of anticancer platinum drugs: evidence that *cis*-diamminedichloroplatinum (II) and *cis*-diammine (1,1-cyclobutanedicarboxlato) platinum (II) differ only in the kinetics of their interaction with DNA. *Cancer Res.*, **46**, 1972.

LEWIS, A.D., HAYES, J.D. & WOLF, C.R. (1988). Glutathione and glutathione-dependent enzymes in ovarian adenocarcinoma cell lines derived from a patient before and after the onset of drug resistance: intrinsic differences and cell cycle effects. *Carcinogenesis*, **9**, 1283.

OZOLS, R.F., OSTECHEGA, Y., MYERS, C.E. & YOUNG, R.C. (1985). High-dose cisplatin in hypertonic saline in refractory ovarian cancer. *J. Clin. Oncol.*, **3**, 1246.

OZOLS, R.F., OSTECHEGA, Y., CURT, G. & YOUNG, R.C. (1987). High-dose in refractory ovarian cancer patients. *J. Clin. Oncol.*, **5**, 197.

STANHOPE, C.R., SMITH, J.P. & RUTLEDGE, F. (1977). Second trial drugs in ovarian cancer. *Gynecol. Oncol.*, **5**, 52.

WILTSHAW, E. & KRONER, T. (1976). Phase II study of *cis*-dichlorodiammineplatinum (II) (NSC-119875) in advanced adenocarcinoma of the ovary. *Cancer Treat. Rep.*, **60**, 55.

WILTSHAW, E. (1985). Ovarian trials at the Royal Marsden. *Cancer Treat Rev.*, **12**(A), 67.

WILTSHAW, E., EVANS, B., RUSTIN, G., GILBEY, E., BAKER, J. & BARKER, G. (1986). A prospective randomised trial comparing high-dose cisplatin with low-dose cisplatin and chlorambucil in advanced ovarian carcinoma. *J. Clin. Oncol.*, **4**, 722.

WOLPERT-DEFILIPPES, M.K. (1980). Antitumour activity of cisplatin analogs. In *Cisplatin. Current Status and New Developments*, Prestayko, A.W., Crooke, S.T. & Carter, S.K. (eds) p. 183 Academic Press: London.

YOUNG, R.C., VON HOFF, D.D., GORMLEY, P. *et al.* (1979). *Cis*-dichlorodiammineplatinum (II) for the treatment of advanced ovarian cancer. *Cancer Treat. Rep.*, **63**, 1539.